# On the Nature of Epoxy Resin Post-Curing

**DOI:** 10.3390/polym12020466

**Published:** 2020-02-18

**Authors:** James C. Moller, Rajiv J. Berry, Heather A. Foster

**Affiliations:** 1 Department of Mechanical and Manufacturing Engineering, Miami University, Oxford, OH 45056, USA; 2 Air Force Research Laboratory, Materials & Manufacturing Directorate, Wright-Patterson AFB, OH 45433, USA; rajiv.berry@us.af.mil; 3 University of Dayton, Department of Mechanical and Aerospace Engineering, Dayton, OH 45469, USA; fosterh1@udayton.edu

**Keywords:** thermosetting resin, cure behavior, computational modelling, cure

## Abstract

Post-curing is intended to improve strength, elevate glass transition, and reduce residual stress and outgassing in thermosets. Also, experiments indicate post-curing temperatures lead to ether crosslinks and backbone dehydration. These results informed molecular dynamics methods to represent them and compare the resulting thermomechanical effects. Diglycidyl ether of bisphenol A (DGEBA)-diamino diphenyl sulfone (DDS) systems were examined. Independent variables were resin length, stoichiometry, and reaction type (i.e., amine addition, etherification, and dehydration). Etherification affected excess epoxide systems most. These were strengthened and became strain hardening. Systems which were both etherified and dehydrated were most consistent with results of post-curing experiments. Dehydration stiffened and strengthened systems with the longer resin molecules due to their intermediate hydroxyl groups for crosslinking. Changes in the concavity of functions fit to the specific volume versus temperature were used to detect thermal transitions. Etherification generally increased transition temperatures. Dehydration resulted in more transitions.

## 1. Introduction

Post-curing is exposure of a cured resin object to temperatures at or above the curing temperature for an extended period. The expected outcomes are increased strength, elevated glass transition temperature, reduced residual stress, and reduced tendency to outgas [1]. Experimental investigations provide insight into reaction kinetics and associated effects of temperature [2,3,4,5] for diglycidyl ether of bisphenol A (DGEBA) epoxy-based resin formulations. Additional experimental investigations provide insight into the relations among thermal processing and the resulting mechanical and physical properties [6,7,8,9,10].

**Figure 1:**
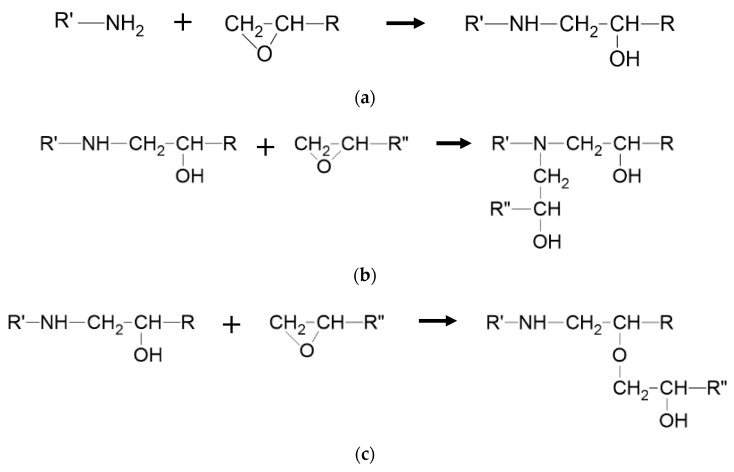
Models of amine addition to form secondary (**a**) and tertiary amine (**b**). Etherification is depicted in (**c**).

The principal reaction among epoxide-bearing and amine-bearing molecules under typical curing conditions is amine addition (depicted in Figure 1). However, additional reactions can occur at significant rates under certain circumstances. The principal of these is etherification (shown in Figure 1c). Depending on where the side group is located, this reaction may or may not occur in the vicinity of an existing crosslink.

DeBakker [4] and Xu and Schlup [5] show that, at typical curing temperatures, etherification generally follows amine addition. Xu and Schlup compared etherification and amine addition rates in tetraglycidyl diaminodiphenylmethane/methylaniline (TGDDM/mAnil) systems. For a stoichiometric blend, added ether link concentration was an order of magnitude less than hydroxyl groups formed by epoxide opening. For excess epoxide formulations, however, ether concentration became significant once reactive amines were exhausted. Epoxide concentration declined in correspondence with ether concentration. Sensitivity of etherification rate to temperature was also established. A near five-fold increase in etherification-to-amine addition rate ratio occurred as temperature increased from 137 to 187 °C. Similar results were reported by Riccardi and Williams [11].

**Figure 2:**
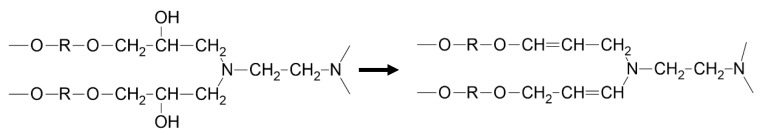
The dehydration model inferred by Grassie et al. [3].

Investigations in thermal degradation of cured epoxies indicate resin backbone dehydration is the most facile reaction. Grassie et al. [3] examined degradation of cured DGEBA epoxy by analyses of volatiles. They concluded water was generated due to autocatalytic interaction among adjacent hydrogen and hydroxyl side groups. The structure change is depicted in Figure 2 where side group removal leads to carbon double backbone bonds. Additional evidence is mass loss from cured samples in thermogravimetric analyses (TGA) at low temperature [3,12,13]. Cheng [12] found losses as much as ten percent when temperature reached 200 °C from samples cured at 140 °C. 

The effects of post-curing on properties has also been explored. Gupta et al. [6] examined the effects on mechanical properties of low-molecular-weight DGEBA epoxy-meta phenylene diamine (mPDA) formulations. For formulations ranging from stoichiometric to 100 percent excess epoxide, post-curing generally decreased tensile modulus. Glass transition temperature over the entire formulation range (100% excess epoxide to 170% excess amine) increased. The increase was distinctly larger (approximately 20 °C) for excess epoxy than for stoichiometric and excess amine blends. Strength was not altered in a distinguishable way. Post-curing dramatically increased crosslink density in excess epoxy cases. The highest crosslink density was a post-cured sample having an intermediate level of excess epoxy (10 parts per hundred of resin). 

Post-curing also reduces density [6,14,15]. Gupta et al. [6] found density decreased on the order of 0.01 g/cm^3^ over the entire formulation range. Kong [14] found a decrease of 0.06 g/cm^3^ in a TGDDM/DDS-based formulation. These were speculated to be due to evaporation of low-molecular-weight contaminants including water. 

Insight into the relationships among chemical structural changes resulting from amine addition, etherification, and backbone dehydration and bulk properties in similar thermoset resins is investigated here via molecular dynamics (MD). The forgoing discussion provides evidence ether crosslinking and dehydration can be significant under conditions consistent with post curing. While curing temperatures are often held at levels for which amine addition predominates, post curing temperatures (having levels reported from 170 to 230 °C [2,6,7,8,9,10,16]) are sufficient to accelerate ether crosslinking and dehydration. 

Ether crosslink addition and dehydrative side group removal in atomistic systems was informed by these experiments. The aim is to augment the inferences about molecular structure derived from experiments with atomistic results to more conclusively identify connections among property and structural changes.

MD-based approaches offer advantages over classical laboratory methods in that confounding factors such chemical reactions in the midst of testing to determine temperature-dependent material properties as well as simultaneous evidence of multiple phenomena can be removed or segregated. Further, test samples can be examined for responses over a wider temperature range than is physically feasible for the material and large samples of molecular configurations can be gathered. 

We know of no reports of simulation of epoxy network formation by amine addition followed by etherification and dehydrative degradation. There have been reports of atomistic simulations of crosslinking which include specific bond formation to create secondary and tertiary amines [17,18,19,20,21,22,23,24,25,26,27,28,29,30,31], bond formation among other specific molecular group pairs [32,33,34,35,36,37,38,39,40,41], link formation among coarse-grain entities [42], special bond reactions [43], and simulated multiple selected reactions based on known kinetic rates [44,45].

Because ether crosslinks differ from amine bonds, the resulting molecular network differs and its respective bulk properties are expected to also differ. Removal of hydroxyl and hydrogen side groups and insertion of carbon–carbon double backbone bonds are expected to alter cured network stiffness and free volume distribution. Due to the presumed countering effects of these molecular changes on bulk mechanical behavior, it is challenging to anticipate the net effect without methodical simulation. 

## 2. Methods and Procedures

The in silico approach involved several steps. Cells were populated with molecules to be reacted. Once equilibrated, dynamics was performed to insert amine and then ether or amine crosslink bonds. Subsequently, adjacent hydrogen and hydroxyl side groups along the resin backbone were decimated and the associated carbon–carbon bonds were increased in order. Systems from each stage were re-equilibrated and probed for free volume. Cells were deformed at constant strain rate in generalized plane stress to obtain mechanical properties. To determine thermal transitions, specific volume was monitored while systems were thermally cycled. 

**Figure 3:**
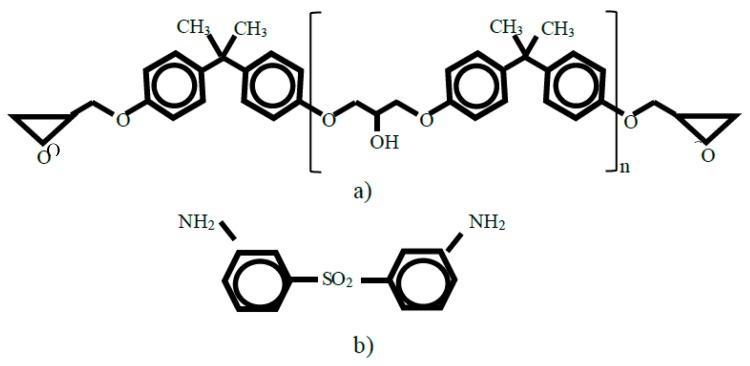
Structural diagrams of diglycidyl ether of bisphenol A (DGEBA) (**a**) and 3,3′ diamino diphenyl sulfone (3,3′ DDS) (**b**).

Systems were constructed by randomly populating cells with unreacted molecules having the intended proportions of amine and epoxide groups. The ingredients (depicted in Figure 3) were 3,3′ diamino diphenyl sulfone (3,3′ DDS) and DGEBA having polymerization indices (*n*) of either zero or one. Systems were large enough that artificial effects of periodic boundary conditions were comparatively small. As shown in Table 1, the total number of atoms was approximately 30,000 in each case. At 1 atmosphere and 298 °K, cell dimensions (ca. 70 Å) are large with respect to the mean unreacted molecule length that the number of cross-links in each cell would range from several hundred to one thousand. Atom interactions were as specified with the Transferable, Extensible, Accurate and Modular (TEAM) force field (Aeon Computational Chemistry). 

During dynamics, likely reactions were found by measuring proximity among all candidate pairs at regular intervals. To represent amine addition and etherification consistent with experiments, crosslinking proceeded in two stages. First, when a primary or secondary nitrogen amine was within a specified search radius of an epoxide carbon, the molecular database was reconstituted to reflect a new carbon–nitrogen bond and the removal of a hydrogen atom from each group. Subsequently, amine addition and ether crosslinking were allowed. The logic for ether bond insertion was similar. Bond and non-bond energy in the immediate vicinity of the new bond was minimized prior to subsequent dynamics. The search radius for progressively increases for each crosslinking stage. In order to increase the likelihood for reactive pairs diffuse into proximity over the comparatively short simulated physical time, dynamics were performed at high temperature (698 °K). 

The limit of curing by amine addition was deemed to have been reached when the search radius became several times larger than the C–N bond length and no new crosslinks were created. Due to diffusional barriers presented as the crosslinked molecular network gels and cures further, additional reactions are highly unlikely. In stoichiometric systems, this resulted in an extent-of-cure of approximately 90 percent. FTIR-based investigations indicate similar extents [2]. Subsequently, etherification reactions were permitted to occur. Similarly, a progressively increasing search radius was applied between dynamic simulations until the evidence indicated all probable reactions had been represented. 

Once amine and ether crosslinks had been created, remaining adjacent hydrogen and hydroxyl groups along resin backbones were decimated to represent the result of dehydration. Charges on remaining atoms were readjusted utilizing the Gasteiger method [46,47]. The associated carbon–carbon bonds became double and the Lennard–Jones parameters were changed to those for double-bonded carbon. Systems were again re-equilibrated. 

Each crosslinked system (i.e., each initial system in Table 1 cured by amine addition, etherified, and dehydrated) was given randomized initial velocity distributions at 1000 °K and then cooled to 50 °K. Initial randomization was repeated five times for each system. During each cooling run, snapshots were archived at 50 °K increments. Each snapshot at 300 °K was used as the initial system state for additional dynamics to obtain equilibrium. The criterion used to deem the system as being in equilibrium was that mean cell volume averaged over 2.5 ps changed by 0.25% or less over a span of 25 ps. 

Glass transition temperature is commonly inferred by cooling at constant rate and pressure and recording the corresponding specific volume as a function of temperature. When it is assumed only one transition can be detected, an approach is to extrapolate from the high- and low-temperature regimes over which specific volume varies linearly with temperature and find the intersection point. The temperature at which the extrapolations intersect is termed the glass transition temperature. Between these regimes, specific volume generally varies nonlinearly. When this is done with MD-based data, the gap in temperature among the regimes can be several hundred degrees Kelvin. Unlike physical tests, MD simulations can be performed over a wide temperature range. This range can be large enough that molecular motions can be frozen at low temperature and can be made active at sufficiently high temperate. 

It is also commonly accepted (and demonstrated when sensitive devices such as dynamic mechanical analyzers are used), however, that complicated molecular structures such as cured epoxy display multiple transitions as the thermal energy level changes. Experiments show multiple transitions occur in epoxies over ranges of this magnitude. Cured DGEBA epoxy has been shown (via dynamic mechanical analysis) to have gamma, beta, and alpha transitions [48,49,50]. Further, it has been shown that multiple transitions of a given type (e.g., beta) can be detected at a succession of temperatures. Careful measurement by the thermally-stimulated discharge method [51] detected multiple gamma and beta transitions over the range −170 to 0 °C in a diglycidyl ether/aromatic diamine system. If multiple transitions are presumed, then, multiple temperature regimes (i.e., multiple ranges of temperature over which the dependence of specific volume on temperature is constant) are anticipated. 

Given this evidence, it was presumed multiple transitions could exist. Because feasible cooling rates are high in MD, it was anticipated undercooling would occur as the sample completed a given transition. Each system was therefore alternately heated and cooled through several cycles at constant pressure (0 atm) while recording specific volume. Heating and cooling rates were 6.25 × 10^−5^ °K/fs. The constancy of the thermal expansion at the peak temperature (1000 °K) indicated all motions were active and systems therefore would be re-randomized before commencing with the subsequent cooling. There were 30,400 specific volume records were collected for each cycle. Data gathered during heating was segregated from cooling to isolate differences in system barrier crossing as energy was added or removed. The local slope and concavity (i.e., second derivative) of the data ensemble for specific volume as a function of temperature were computed. Slope was used to identify the range over which transitions were occurring. When there was a distinct peak in the concavity, this was taken to represent a transition. Data from several cycles was used to establish confidence intervals for slope and concavity. At extremely low or high temperature, slope was expected to be insensitive to temperature change. 

To measure and compare strength and stiffness, several types of stress states (i.e., biaxial tension, uniaxial tension, pure shear, and uniaxial compression) were applied to each system. In each case, the system was deformed at a constant effective strain rate of 10^8^ s^−1^. This was accomplished by applying a succession of dynamics simulations. Stress values were kept in the proportion to match the chosen stress state and adjusted to maintain constant strain rate [52]. 

Each response (i.e., von Mises stress as a function of effective strain) was fitted to a polynomial. The yield strength was taken to be either the first maximum in the function or the first inflexion point, whichever occurred at a lower effective strain. Elastic modulus was taken as the slope of the fit evaluated at zero strain. 

**Figure 4:**
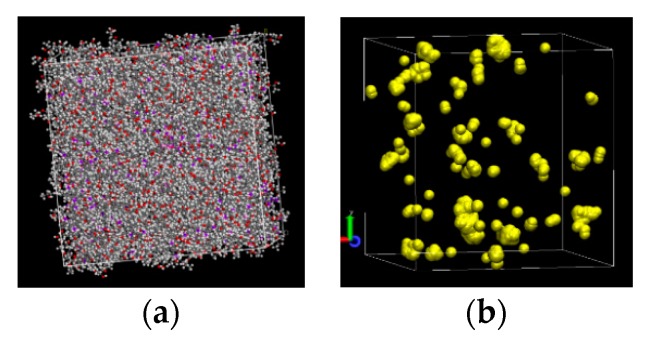
Renderings of a cell with atoms (**a**) and the corresponding probe spheres occupying free space (**b**).

The amount and distribution of free volume were estimated with a probe sphere approach. Given the nuclear locations and van der Waals radii of each atom, probe spheres were repeatedly randomly placed in the given cell. Each sphere was tested for overlap with any atom or any free space already identified. A non-overlapping sphere was treated as discovered free volume. Probing began with 4 Å radius spheres. Once a set number of attempts had been reached, searching was deemed exhausted and incrementally smaller probe spheres were used. Example representations of the atoms and free volume in a cell are shown in Figure 4.

## 3. Results and Discussion

**Figure 5:**
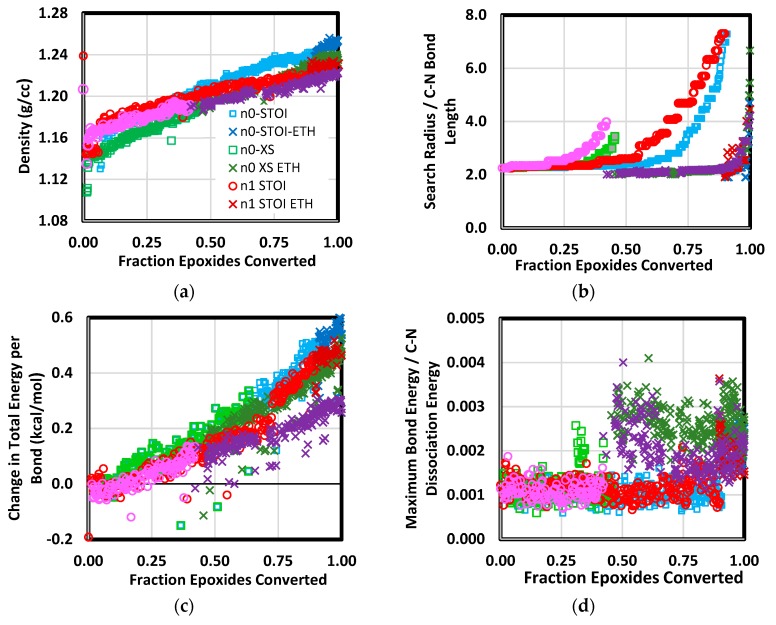
Quantities tracked during crosslinking simulation. Depicted are density (**a**), group–group search radius (**b**), change in total energy per bond (**c**), and maximum bond energy normalized by C–N bond dissociation energy (**d**). Labels including “ETH” correspond to the crosslinking stage in which both ether and amine crosslinks were formed. Labels “STOI” and “XS” correspond to stoichiometric and excess epoxide blends, respectively.

Several quantities were recorded to monitor the nature of crosslinking by amine addition and subsequent etherification. As shown in Figure 5a, all systems densified during amine addition and etherification. The rate of increase with respect to the fraction of epoxides converted was approximately the same for all systems and the same between amine addition and etherification. The short-chain (i.e., *n* = 0) systems had the largest final density. As stated earlier, crosslinking proceeds by incrementally increasing a search radius for reactive groups. Initially, the number of epoxides converted by amine addition increased rapidly for a search radius just over twice the equilibrium C–N bond length (as in Figure 5b). Following this period, the search radius steadily increased for small increments in the fraction converted. Once the no new reactive groups were found over the intervening dynamics simulation increment, ether crosslinks were allowed. At this point, numerous additional epoxides were converted to ether crosslinks due to their proximity to hydroxyl groups. In reality, crosslinking proceeds because it is energetically favorable at the given conditions and species concentrations. The simulated crosslinking leads to a comparatively minor increase in energy. As shown in Figure 5c, the increase in system energy on a per-bond basis is less than 1.0 kcal/mol. As shown in Figure 5d, the maximum bond energy was consistently less than one percent of the C–N bond dissociation energy. 

**Figure 6:**
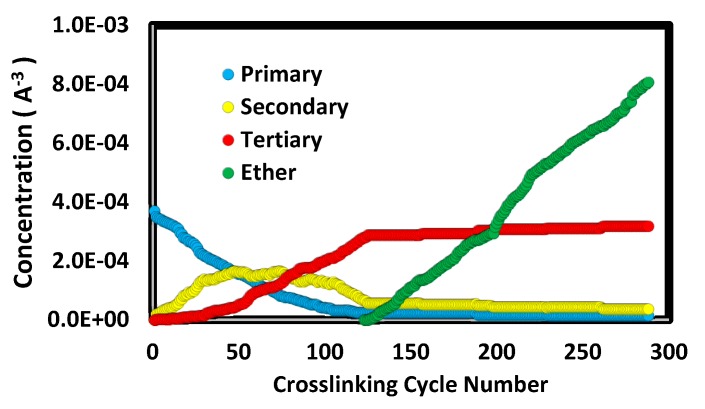
Amine and ether concentrations as a function of crosslink cycle number for the long-chain (*n* = 1) excess epoxide system. The ether concentration is the increased over the initial uncured system.

The progression of relative concentrations of amine types and ether increase during crosslinking is similar to findings from experiments [4,5]. Results for the long-chain (i.e., *n =* 1) excess epoxy system are shown in Figure 6. The initial consumption of primary amines is principally associated with secondary creation. Once approximately half the primary amines are consumed, the secondary concentration reaches a maximum and there is steady growth in tertiary amine concentration. Once the etherification criterion is met, the added ether concentration rises until nearly all epoxides are consumed.

**Figure 7:**
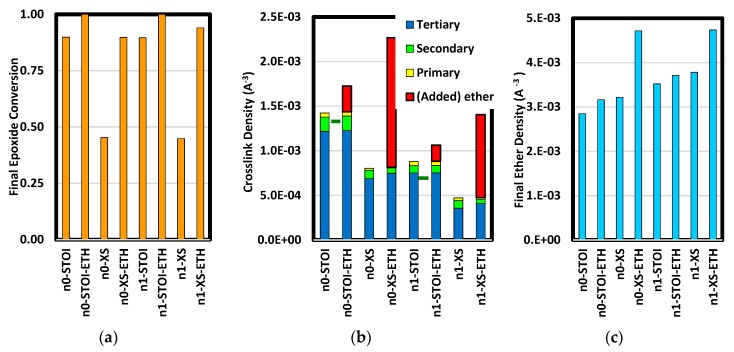
Results of amine addition and etherification on epoxide conversion (**a**), crosslink density (**b**), and total ether density (**c**). The green fiducial marks in (**b**) are values measured by Glad and Kramer [53] for amine-cured systems using methylene diamine as the curing agent.

Demographic results from amine addition and etherification enable comparison of degree-of-cure, crosslink density, and amine-to-ether crosslink proportion. Degree-of-cure is defined here as the fraction of epoxides converted. In Figure 7a, stoichiometric systems went to full conversion when etherified. While excess epoxide systems without etherification had the lowest degree-of-cure, the proportion more than doubled when etherified. Apparently, diffusion constraints presented by these networks late in curing precluded full conversion. A variety of crosslink densities were attained (shown in Figure 7b). A high level was attained with a short resin molecule at stoichiometric proportions. The highest occurred in the etherified short-chain excess epoxide system. Intermediate levels occurred in the short-chain excess epoxide system which had not been etherified and the long-chain stoichiometric formulation. Ether backbone groups, be they due to the original resin or the result of etherification, are a significant part of the structure. As such, these play a significant role in the network geometry, stiffness, and strength. As shown in Figure 7c, the ether density is greater than the crosslink density in all cases and is largest in etherified excess formulations. Final amine group concentrations indicate additional curing in excess epoxide cases (Figure 7b). In stoichiometric cases, amine addition led to diffusional impediments to further amine-epoxide reaction that were not overcome during etherification. In excess epoxide cases, the low crosslink density at the end of amine addition allowed enough network porosity for more significant diffusion. 

**Figure 8:**
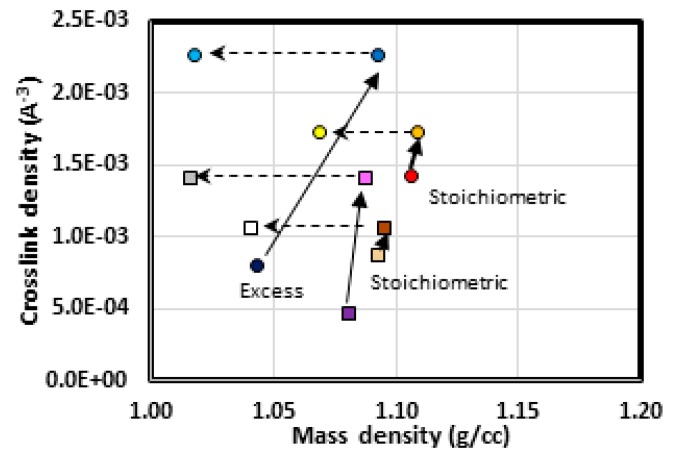
Crosslink density as a function of mass density. Changes due to etherification and dehydration are indicated by a solid and dashed arrows, respectively. Short- and long-chain resins are depicted by circles and squares, respectively. Stoichiometric and excess epoxide formulations are indicated.

Mass density, stiffness, and strength are commonly found to correlate with crosslink density. Etherification was here to distinctly increase crosslink density (especially in excess epoxy cases) and increase in density. While the simulated dehydration did not alter the crosslink density, density changes were generally more distinct than those from etherification. The respective values and density changes are depicted in Figure 8. For all formulations, the mass density increase associated with etherification was not as large as the decrease resulting from dehydration. The largest density increase upon etherification was in the short-chain excess epoxide formulation. This case also had the largest number of crosslinks added by etherification. The overall drops in density (between amine-cured to dehydrated states) ranged from 0.025 to 0.065 g/cc. Both the observation of a mass density decrease and the magnitude of the decreases are consistent with findings from experiments [6,14]. 

Mass loss due to complete dehydration was consistent with experiments. Kong [14] reported a density decrease of 0.06 g/mm due to post-curing. For the cell volumes considered here, the equivalent mass loss would have been 2.10 × 10^−20^ g. Masses removed from etherified systems are listed in Table 2. Mass loss estimates are only applied to etherified systems because dehydration is taken to be a result of post-curing. Not only are the results within a factor of two of the mass loss inferred from the experiments, the trends are in agreement with those of Gupta et al. [6]. Their results indicated mass loss was larger from stoichiometric systems than excess epoxy systems. 

The amount of overall decrease in mass density does not directly correlate with the amount of mass lost to dehydration. The short-chain excess epoxide system had the smallest mass loss and smallest mass density decrease. This system also had the largest total crosslink density. The systems with the largest mass loss and largest mass density decrease were the long-chain stoichiometric and the long-chain excess epoxide formulations, respectively. The long-chain stoichiometric formulation has the lowest total crosslink density after etherification. The long-chain excess epoxide formulation has the lowest amine crosslink density. It therefore appears mass loss scales inversely with crosslink density and that amine crosslink density is inversely related to mass density decreases. 

**Figure 9:**
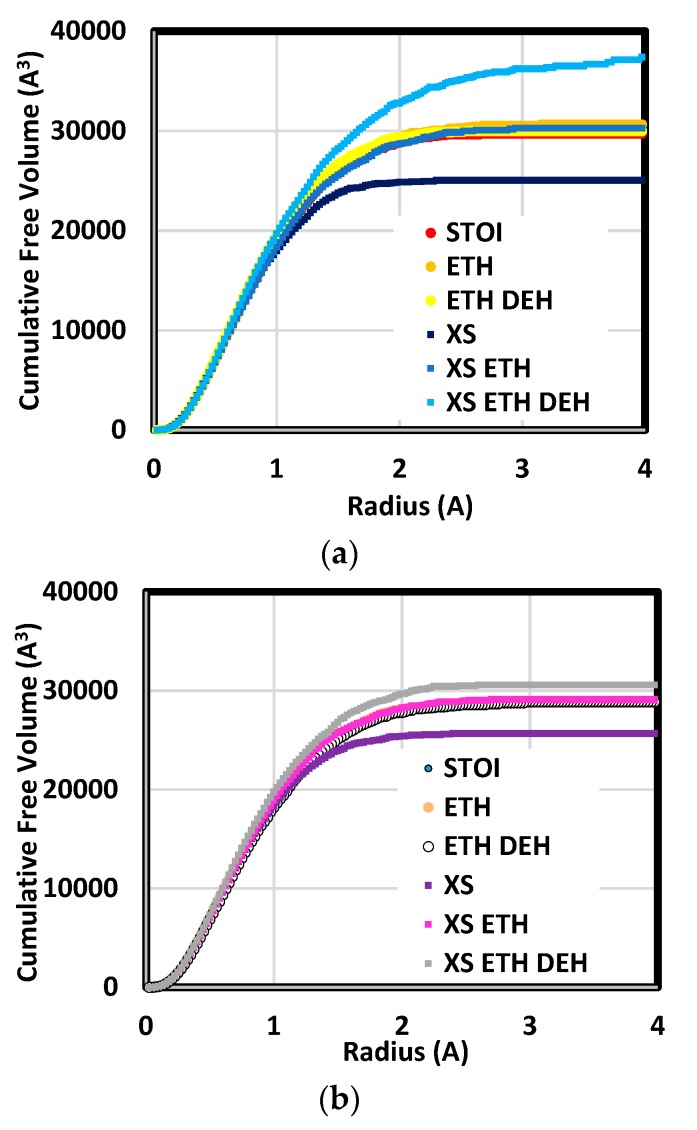
Cumulative free volume distributions for short (**a**) and long (**b**) chain systems.

Small species diffusion rate and segmental motion frequency depend, in part, on free volume distribution [54]. Further, it has been demonstrated epoxy fracture toughness and shear modulus are functions of free volume fraction [55]. Results (shown in Figure 9) are presented as cumulative free volume as a function of radius. At a given radius, the cumulative free volume is the total free volume at this radius and smaller. Comparison among distributions reveals quantities and hole sizes which are more prevalent in each system type. Holes larger than 2.5 Å generally were not found. The exception was the short-chain system which had been etherified and dehydrated. This system included hole radii as large as 3.95 Å. 

Etherification and dehydration had the most distinct effect on free volume in excess epoxide systems. For both resin chain lengths, the total free volume in these systems was comparatively low following amine addition (labelled XS in the plots). Etherifying these resulted in large increases in crosslink density with a small decrease in mass density. Associated with the mass density decrease is an increase in the number of holes principally in the 1.0–2.5 Å range and a free volume increase of approximately one percent of the cell volume. Subsequent dehydration led to an increase in the number of holes in the ranges 0.8–4.0 and 0.8–2.3 Å in the short- and long-resin cases, respectively. Based on this result, it is inferred that increasing backbone carbon bond order at dehydration sites led to molecular structures which were locally stiff and strong in the vicinity of free volume and therefore resisted collapse.

The effect of dehydration on free volume distribution was distinctly different for stoichiometric blends. In these, distribution changes following etherification and dehydration were almost undetectable. These systems had a much lower ether crosslink density and a high amine crosslink density. In that amine addition does not reduce the number of hydroxyl groups, there were a large number of sites for dehydration. Further, oxygen has a comparatively large molecular weight given its van der Waals radius. This means the density decrease due to dehydration in stoichiometric formulations is principally due to removal of a comparatively large number dense atoms. Unlike the excess epoxide cases, the small change in total free volume means the structure in the vicinity of dehydration sites was complaint enough to fill the voids created. 

These distributions show generally finer hole sizes than those reported by positron annihilation lifetime spectroscopy (PALS) [55,56,57,58,59]. Where PALS measurements of cured epoxies have indicated a peak hole radius of ca. 2.5 Å, the most common probe radius here was consistently 0.5 Å. This difference may originate from the capability of PALS instruments and the underlying theory used to infer hole radius. The mean free volume hole radius is calculated from ortho positronium (o-Ps) annihilation time using the Tao–Eldrup model [60,61,62]. This model presumes the o-Ps interaction with its surroundings is described by a spherically symmetric potential well of radius $R_{\infty }$ with a border layer of thickness δ. Calibrations done with gasses and liquids (not polymers) have shown the border thickness to be 1.66 Å. Correspondingly, the smallest detectable hole via PALS is reported to be ca. 1.7 Å [63].

In addition to being related to toughness and strength, free volume also affects small-species diffusion rate and moisture uptake. The effects of thermal processing on these have been investigated via experiments. Aronhime et al. [64] found curing decreased diffusion coefficient and increased water uptake. The latter outcome was attributed to increased free volume. The former was attributed to the increasingly rigid network as curing proceeded. Belton [65], however, found post-curing led to increases in both diffusion coefficient and moisture uptake. These were attributed to volume recovery during aging and increased sample defect volume. The results reported here for excess epoxide blends are consistent with these and help substantiate the assertions of those researchers. Dehydration led to distinctly increased free volume where much of the increase was due to the appearance of comparatively large holes. 

**Figure 10:**
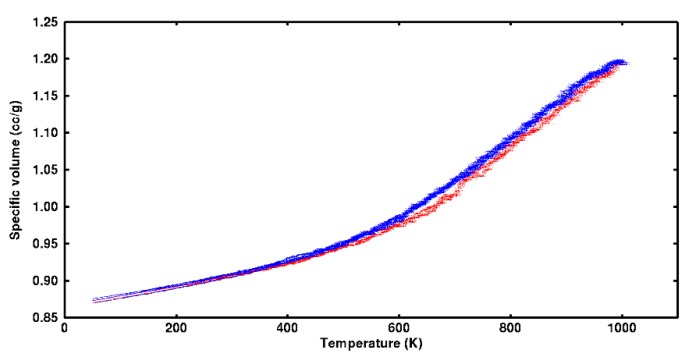
Specific volume as a function of temperature for the short-chain stoichiometric system. Data points for five cycles of heating and cooling are blue and red, respectively.

Thermal cycling applied to each system showed there were statistically significant differences in specific volume among heating and cooling over most of the test temperature range (50–1000 °K). Specific volume data over five cycles for one system are plotted in Figure 10. Detectable differences among heating and cooling are found at temperatures as low as 350 °K. Specific volume on heating was generally lower than during cooling. This difference is attributed to the undercooling required to cause segments and side groups to nest in lower energy wells and take on more compact conformations as heat was removed. 

Previous experiments provide insight into the effects of amine and ether crosslinks on thermal transitions as well as guidance for the temperature range to analyze. Rudnev and Oleinik [51] used thermally-stimulated-depolarization to identify transitions to high resolution and found multiple *β* and *γ* relaxations. They found two *β* peaks between 173 and 233 °K attributed to backbone hydroxether motions. They speculated *γ* peaks (occurring between 113 and 173 °K) were associated with phenyl ring motions. Pogany [48] examined mechanical relaxation peak shifts in a stoichiometric bisphenol A resin/diethylene triamine blend. Glass transition was near 413 °K and *β* relaxations between 323 and 373 °K lost prominence as curing proceeded. A *γ* peak shifted from −60 to −40 °C as curing proceeded. Ochi et al. [49] examined low-temperature relaxations in cured epoxy systems containing various combinations of amine and ether crosslinks. They found *γ* and *β* relaxations in the vicinity of 133 and 213 °K, respectively. When tan *δ* was compared among the systems, they concluded the *γ* relaxation would occur when the backbone included four or more methylene mers in the backbone. They also found the *β* relaxation was 30 °K lower in ether-crosslinked systems compared to amine-crosslinked systems. By examining the dependence of tan *δ* peaks on degree of cure, they concluded *β* relaxations are associated with hydroxyether [–CH_2_–CH(OH)–CH_2_–O–] group motions in amine-crosslinked systems and that a different group motion is associated with *β* relaxation in ether-crosslinked systems. 

**Figure 11:**
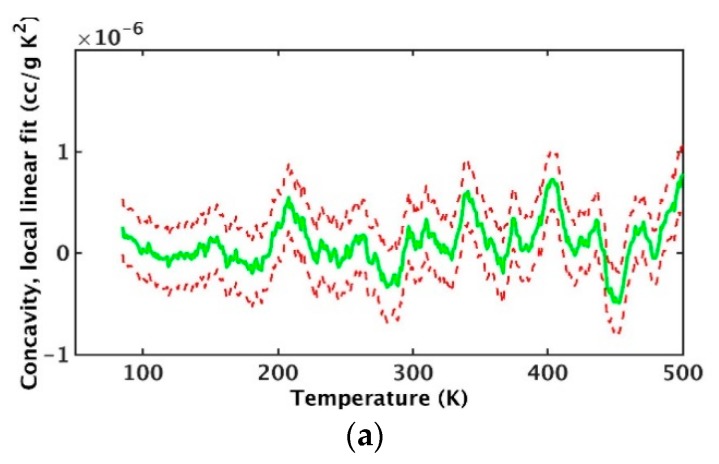

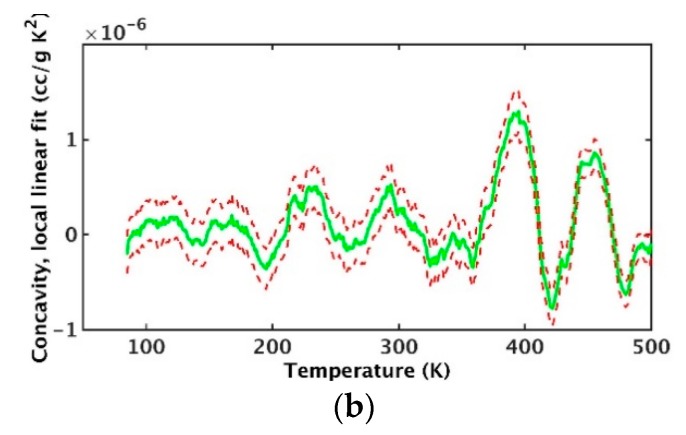
Local concavity versus temperature for short-chain etherified system for heating (**a**) and cooling (**b**). Dashed lines are the 95 percent confidence interval.

Given that physical experiments indicate transitions occur below 500 °K, data from 75 to 500 °K were analyzed for concavity peaks in specific volume as a function of temperature. Cooling data were used because undercooling resulted in distinct drops over narrow temperature ranges. At each temperature (75 to 500 °K in increments of 1 °K), a second order polynomial was fit to the data over a 50 °K range centered at the respective temperature. An example illustrating the significance of concavity variations is provided in Figure 11 which shows the results for the succession of fits at 1 °K intervals as well as a band representing the associated 95 percent confidence interval. The confidence interval is consistently less than the amount of variation in concavity. Further, peaks are more prominent for cooling than for heating. 

**Figure 12:**
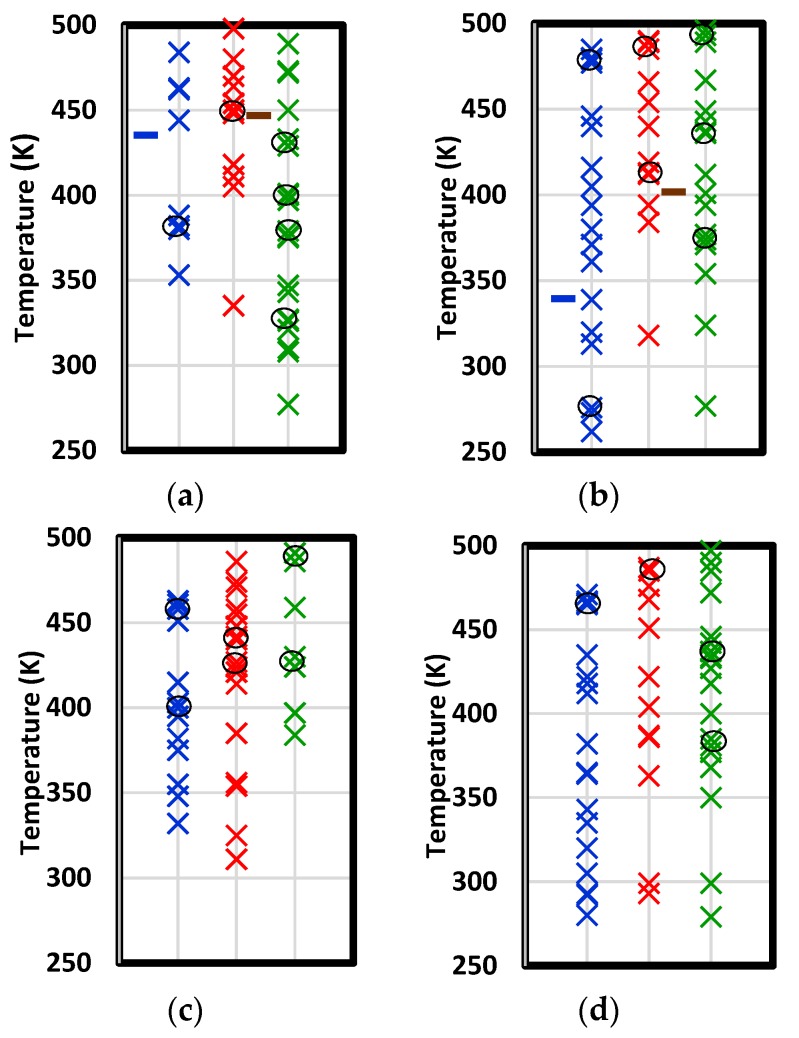
Temperatures of concavity peaks in the specific volume versus temperature plot upon cooling for short-chain stoichiometric (**a**), short-chain excess epoxide (**b**), long-chain stoichiometric (**c**), and long-chain excess epoxide (**d**) formulations. Symbols are colored according to: amine addition only (blue), amine addition and etherification (red), and subsequent dehydration (green). Points were obtained from five thermal cycles. Instances of least three peaks within a range of 10 K are circled. Fiducial marks in (**a**) and (**b**) are the measured T_g_ values of Gupta et al. [6] for cured (blue) and post-cured (brown) specimens.

When concavity peaks obtained from a succession of thermal cycles are logged and compared, evidence of the effects of stoichiometry, resin chain length, etherification, and dehydration on transitions emerge. In Figure 12, narrow temperature bands in which concavity peaks frequently occurred are indicated by ovals. 

In three of four formulations, more transitions are detected in the dehydrated state than in the amine-cured or etherified states. Two of the references determined some transitions are associated with hydroxether segments. According to the model presented here, such segments would be liable to dehydration. Consequently, the local backbone stiffness would change and free volume surrounding the segment would be expected to change. 

Fiducial marks indicating the glass transition temperatures measured by Gupta et al. [6] for the same short-chain system are shown in Figure 12a,b. They found post-curing increased the glass transition. The increase was larger for excess epoxide formulations. When compared with the transitions inferred here, the stoichiometric formulation (shown in Figure 12a) has one transition in the amine-cured state and four in the dehydrated state. Two of these are at temperatures greater than that of the amine-cured transition value. In the sort-chain excess epoxide formulation (shown in Figure 12b), each system has one transition greater than 475 K. The change in the measured glass transition due to post-curing is similar to the change in the average transition between the amine-cured and dehydrated cases. Given the evidence cited of multiple transitions [51], Gupta may have obtained a glass transition that reflected multiple relaxations enabled over a relatively narrow temperature range. 

**Figure 13:**
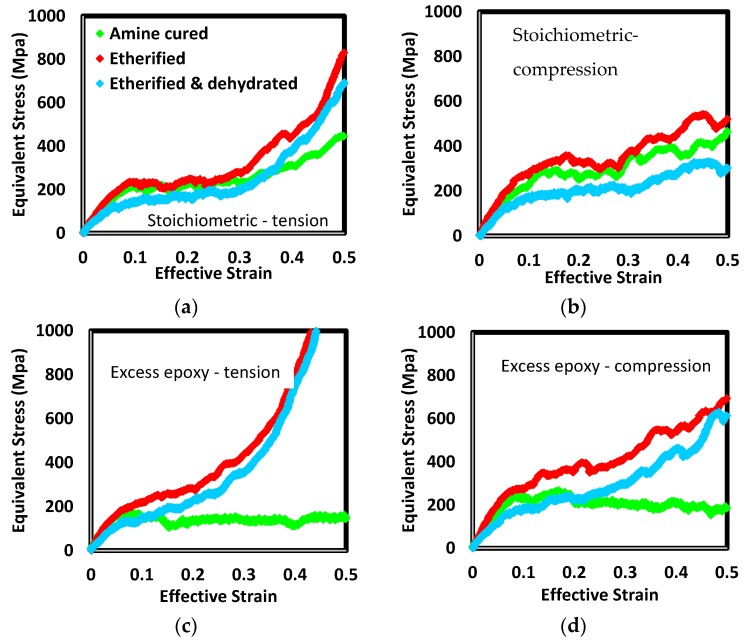
Equivalent stress verses effective strain for uniaxial tension (**a**, **c**) and compression (**b**, **d**) for short-chain resin at constant effective strain rate. Stoichiometric and excess-epoxide formulations are presented in (**a**, **b**) and (**c**, **d**), respectively.

**Figure 14:**
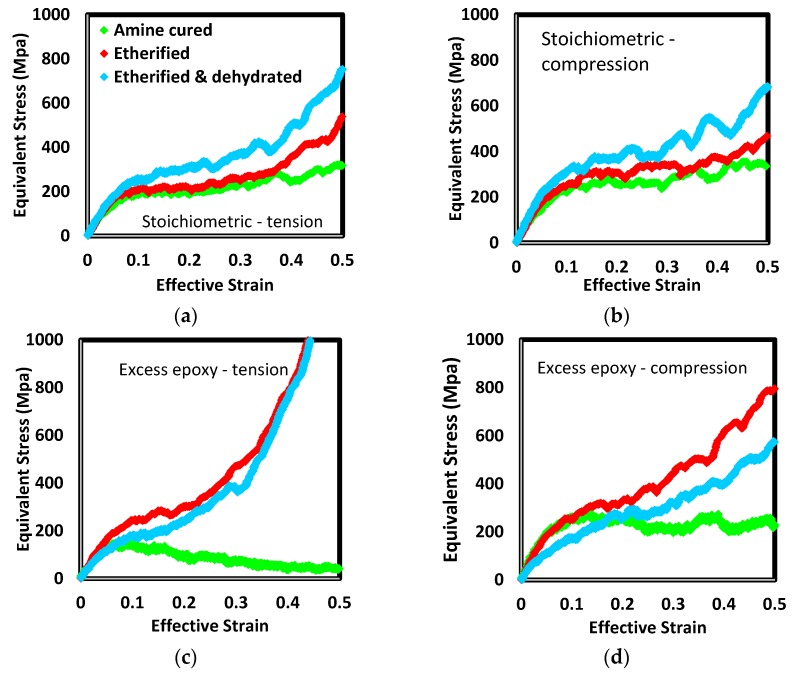
Equivalent stress verses effective strain for uniaxial tension (**a**, **c**) and compression (**b**, **d**) for long-chain resin at constant effective strain rate. Stoichiometric and excess-epoxide formulations are presented in (**a**, **b**) and (**c**, **d**), respectively.

The effects of each processing step, resin chain length, and the sign of the stress in uniaxial loading (i.e., tension versus compression) are illustrated in Figure 13 and Figure 14. The method for loading the samples is described in [52]. A plane stress state (e.g., biaxial tension) and strain rate is selected. That stress state is applied for a series of brief periods of dynamics. At the end of each period, the effective strain is computed and compared with the target value for the selected strain rate. Depending on the level of error among the actual and target strains, the stress magnitude is adjusted for the upcoming period. For a given system and state (e.g., short-chain stoichiometric cured by amine addition only), compression generally resulted in higher strength and less strain hardening. In excess epoxide formulations with only amine crosslinks, systems remained plasticized to the extent that they were perfectly plastic (e.g., short-chain tension or long-chain compression cases) or strain softening (e.g., short-chain tension or long-chain compression cases) following yield. These networks did not have sufficient cross-link density to mechanically diffuse strain energy to less loaded strands.

The most distinct effect is etherification of excess epoxy systems. With no etherification, the response is either perfectly plastic or strain softening. Once etherified, the systems were among the strongest at yield and showed the highest strain hardening rate. 

Etherification alone generally resulted in increased strength and strain hardening. Due to the small number of ether links added to stoichiometric formulations, the amount of increase was detectable but small. In excess epoxide formulations there was a distinct effect of etherification in the post-yield response. Up until the apparent yield point, the responses of amine-cured and etherified versions were essentially identical. Following yield, systems which had displayed perfectly plastic or strain softening behavior with only amine crosslinks now showed strain hardening to extents larger than the corresponding stoichiometric formulations. As shown in Figure 8, etherification of these more than doubled crosslink density and resulted in the highest total ether density of all systems. The lack of added stiffening at small strain from the presence of the additional ethers indicates other parts of the backbone are absorbing strain energy. The added ether links played a significant role once plastic flow was initiated. The force field terms shows angles including amine nitrogen as the central atom are approximately twice as stiff as those including ether oxygen. Further, the potential energy barriers for dihedrals including amine nitrogen as a central atom are an order of magnitude greater than those including ether oxygen. It therefore appears primary and tertiary amines provide small-strain stiffness and, as plastic flow occurs, strands in the vicinity of added ether links uncoil with a small amount of additional work. Once fully uncoiled, the strands associated with them restrict further flow. 

Dehydration generally decreased stiffness and strength compared to values following etherification. Because dehydration did not involve interruption of network strands, the dependence of stress on strain in plastic regions were not dramatically altered. The exception was the long-chain stoichiometric formulation. In this formulation, both stiffness and strength increased. The distinguishing feature of this formulation and the changes due to processing are associated with the hydroxyl group in the middle of the long chain (*n* = 1) resin molecule. This side group is not present in the short chain molecules. In the excess epoxide long chain formulation, these groups are largely involved in etherification. While these become crosslink sites, ether backbone bonds rotate relatively easily. In stoichiometric formulations, however, few of these hydroxyl groups are consumed due to the low density of unreacted epoxides following amine crosslinking. When this stoichiometric formulation is dehydrated, then, there are numerous hydrogen/hydroxyl pairs removed and carbon–carbon bonds increased in order. The doubled bond order increases the resistance of the backbone to torsion. In all the other formulations examined here, such carbon–carbon bond increases occur in the immediate vicinity of amine crosslink sites. In these locations, the network is already stiffened by C–N bonds (which are also comparatively resistant to torsion). 

**Figure 15:**
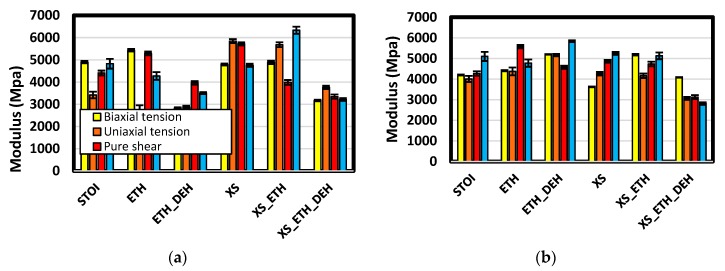
Comparison of zero-strain moduli among various stress states. Charts (**a**,**b**) are results for short- and long-chain systems, respectively.

Comprehensive and quantitative comparison of moduli under several plane stress states provide additional insight into the relations among properties and structure. Moduli in biaxial tension, uniaxial tension, pure shear, and uniaxial compression are shown in Figure 15. Among the discernable relations is that etherification increased modulus in pure shear for stoichiometric formulations but lowered them in excess formulations. In pure shear, the system under load is generally not dilatational. In stoichiometric formulations, there were comparatively few added ether links, whereas the number of ether links added was the highest in the excess formulations. Further, these ether links are generally added in the immediate vicinity of amine crosslinks. It is inferred that such placements led to a more compliant network in shear. 

With the exception of the long-chain stoichiometric formulation, dehydration generally decreased modulus compared to the etherified state. The reason for this exception is again inferred to be associated with the different placement of ether bonds in this case that leads to a better dispersion of crosslinks within the sample volume. In the other formulations, the increased compliance upon dehydration is inferred to be associated with free volume insertion. The most distinct moduli decreases due to dehydration are in excess epoxide formulations, especially under uniaxial compression. Moduli decreases appear to be associated with mass density changes in that excess formulations also underwent the largest decreases in mass density upon dehydration. 

**Figure 16:**
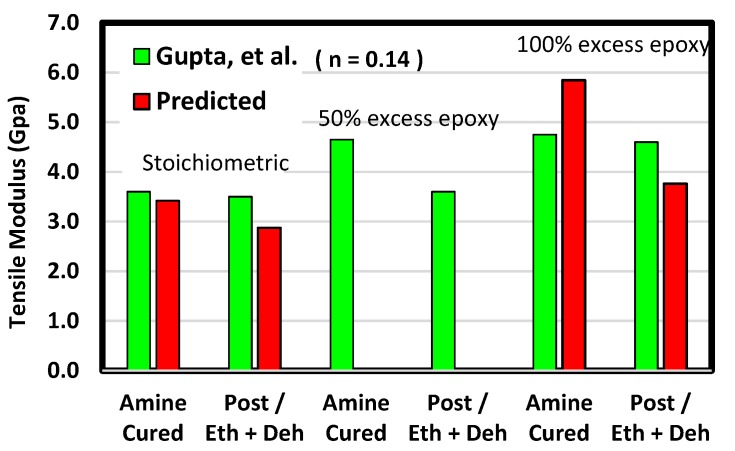
Predicted tensile modulus for the short-chain system compared with the experiments of Gupta et al. [6]. Amine-cured moduli for the same formulation are plotted together. Results from post-curing experiments and predictions including both etherification and dehydration are plotted next to each other.

In the short-chain systems, moduli for excess epoxy formulations were generally larger than for stoichiometric formulations. This outcome is consistent with experiments [6]. Tensile moduli are compared in Figure 16. The results from experiments show post curing decreases the modulus and that the modulus values and the amounts of decrease following etherification and dehydration are similar among both predictions and experiments. Although the test conditions among experiments and predictions differ numerous orders of magnitude in strain rate, agreement was within 20%. 

**Figure 17:**
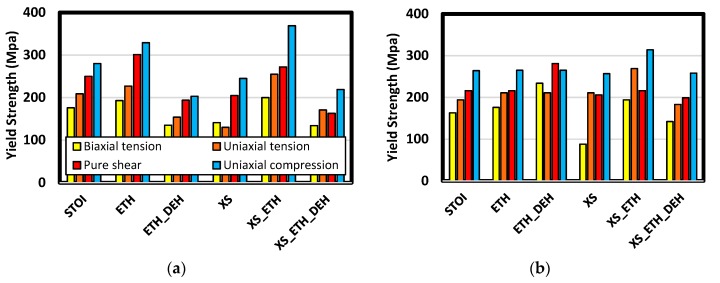
Comparison of yield strength among various stress states. Charts (**a**) and (**b**) are results for short- and long-chain systems, respectively.

Consistent with yield theories often applied to polymers (e.g., Drucker–Prager), yield strength was generally lowest in biaxial tension and largest in uniaxial compression (shown in Figure 17). Etherification led to modest increases in strength in stoichiometric formulations and distinct increases in excess epoxide cases. The largest effect of etherification and highest strengths came in the short-chain excess formulation. This system also had the largest total crosslink density. Dehydration of these, however, resulted in significant strength decreases. Again, there is evidence dehydration had a lesser effect in the long-chain systems.

## 4. Conclusions

Novel methods to represent etherification and dehydration in amine-cured epoxy molecular structures in a molecular dynamics system have been introduced. These were informed by the results of scientific experiments. Such changes to a molecular structure are relevant, for example, in the context of post curing. The result is that predictions are more realistic than the crosslinking strategies heretofore reported in the literature. Further, the results presented here provide additional insight into the nature of post-curing over what is inferred from experiments alone. 

Comparing predictions with experiments, best agreement is observed when etherification and dehydration are included. Evidence from processing experiments indicates both etherification and dehydration occur during post curing. The evidence shows the epoxy–resin blend itself has the capacity to devolve the amount of water consistent with the mass lost in thermogravimetric tests at low temperature ranges.

The results also indicate post-curing is appropriate for improving properties when certain formulations are selected. In particular, resins bearing one or more hydroxyl groups along the backbone appear to have not been compromised when dehydration was applied. 

A novel method for detection of thermal transitions is introduced. It is sensitive to detect evidence of undercooling. Multiple transitions in the vicinity of published glass transition temperatures were found. The effect of post curing on transition is most consistent with the transitions found in simulated systems which had been both etherified and dehydrated. Experiments indicate molecular segments susceptible to dehydration are associated with certain thermal relaxations (and therefore dehydration alters transition temperatures). Consistent with this, predictions showed dehydration had a notable effect on the number and temperature of transitions. 

## Figures and Tables

**Table 1:** Summary of system ingredients prior to crosslinking: 100% excess epoxy and stoichiometric formulations are indicated by “XS” and “STOI”, respectively. Resin molecule degrees of polymerization is designated by “n0” and “n1”.

Name	Resin Chain Polymer-ization Index (n)	Number of DGEBA Molecules	Number of DDS Molecules	Number of Atoms	Original Number of Epoxide Groups	Original Number of N–H Bonds	Number Epoxides/Number N–H Bonds
n0-STOI	0	462	231	31185	924	924	1.0
n0-XS	0	500	125	30125	1000	500	2.0
n1-STOI	1	280	140	30380	560	560	1.0
n1-XS	1	300	75	30375	600	300	2.0

**Table 2:** Predicted mass lost due to complete dehydration of etherified systems.

n	BLEND	Number of OH	Change in Mass (g)
0	Stoichiometric	842	2.80E−20
0	Excess Epoxy	476	1.58E−20
1	Stoichiometric	784	2.60E−20
1	Excess Epoxy	573	1.90E−20
